# Necrotizing Fasciitis of the Cervical Region following Extravasation Injury

**DOI:** 10.1155/2012/941578

**Published:** 2012-11-21

**Authors:** Ayşe Özlem Gündeşlioğlu, Emine Çiğdem Özen

**Affiliations:** Department of Plastic, Aesthetic and Reconstructive Surgery, Meram Medical Faculty, Necmettin Erbakan University, 42090 Konya, Turkey

## Abstract

Necrotizing fasciitis is a rapidly progressive soft tissue infection that can cause local tissue destruction, necrosis, and life threatening severe sepsis. Necrotizing fasciitis in the head and neck region caused by an extravasation injury is rare. This paper reports a patient with necrotizing fasciitis of the cervical region caused by an extravasation injury which required an early surgical debridement.

## 1. Introduction

Necrotizing fasciitis is a rare, potentially lethal bacterial infection which was first described by Hippocrates around the fifth century and the term “Necrotizing fasciitis” was first used by Joseph Jones, a former Confederate Army surgeon, in 1871 [1]. From the clinical point of view, necrotizing fasciitis usually starts as an infection manifested by erysipelas and gradually develops into cellulitis which results in necrotizing fasciitis. Deep soft tissue, including fascia and muscle, is most frequently involved. It progresses rapidly, leading to significant morbidity and mortality. The usual anatomical regions of involvement are trunk, lower extremities, perineum, and scrotum [2]. However, necrotizing fasciitis of the head and neck region caused by extravasation injury is rare. Affected patients are usually neonates [3]. Chemical toxicity, osmotic or pressure effects of extravasated drugs can result in tissue necrosis, fibrosis, or compartment syndrome according to the affected side. Although extravasation injury and the resultant skin necrosis are usually easily recognized and remain localized, severe soft tissue fibrosis may involve the tissues underneath the healthy-looking skin. Underestimation of this necrotic- and fibrotic-scarred tissue may lead to devastating complications such as necrotizing fasciitis or even death, especially in immunocompromised individuals [4].

## 2. Case Report

**Figure 1:**
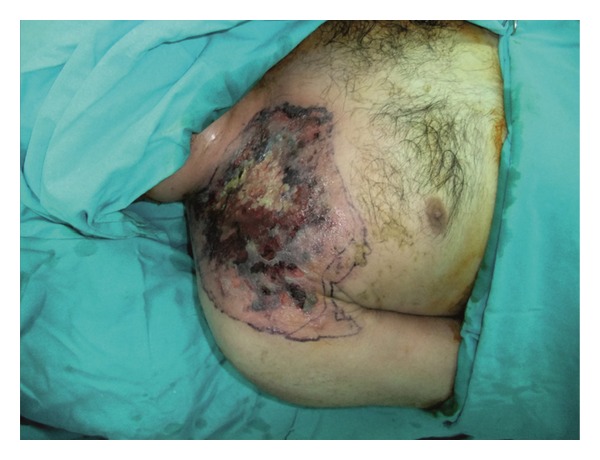
Photograph of the patient showing the signs of necrotizing fasciitis, including necrosis, edema, erythema, and induration on the skin.

**Figure 2:**
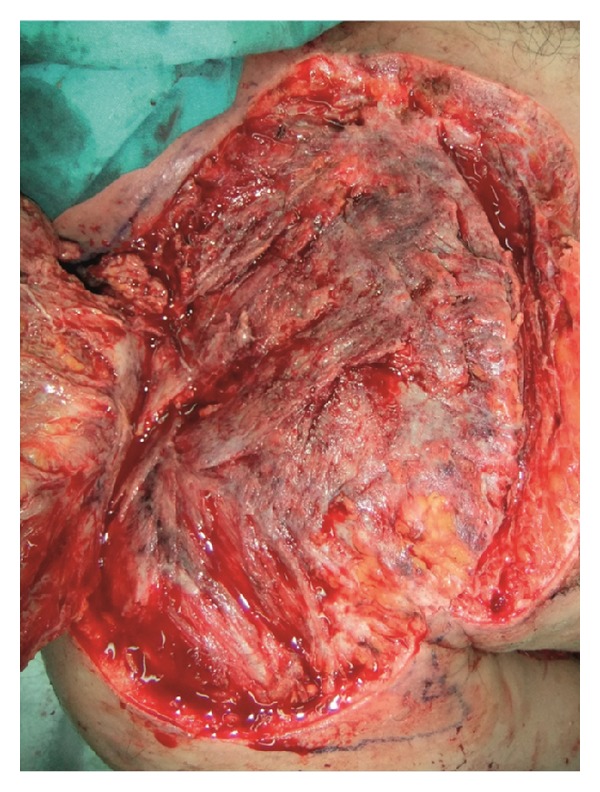
Intraoperative view showing the pectoralis major and deltoid muscles with fibrotic and necrotic areas. All affected muscles were debrided.

A 32-year-old male patient with a history of acute myeloid leukemia presented to Plastic, Reconstructive and Aesthetic Surgery Department from Oncology Department with necrosis, erythema, and edema of the right supraclavicular area extending to the infraclavicular region and the right shoulder (Figure 1). The patient could not move his arm because of severe pain. Two weeks before his present admission, a tunneled central venous catheter had been placed into the right jugular vein by invasive radiology department and he had been treated with antineoplastic drugs such as Cytarabine, Idarubicin via this catheter. Dressing was made with antiseptic solutions around the catheter. The injection site had become erythematous and edematous immediately after injection. After a day, jugular venous catheter had been removed because of the probability of catheter infection. Blood, catheter tip cultures had been obtained and an ultrasound imaging had been performed for the possibility of abscess in injection site. But the results of cultures were negative; there was no abscess in the injection site, no thrombosis in right jugular vein according to ultrasonography, which can be considered as evidence of extravasation injury without infection. The patient had been followed by dressing changes and antibiotics (vancomycin, piperacillin/tazobactam) without any surgical intervention. The day before presentation to us, necrosis and erythema progressed to involve the peripheral regions, and he had severe pain. On the day of presentation, his body temperature was 39.8°C and his blood pressure was 100/60 mmHg. Laboratory findings included a hemoglobin concentration of 8.8 g/dL, a white blood cell count of 4.23 K/uL, and a platelet count of 79 000 e/uL. Levels of procalcitonin and C-reactive protein were 7.9 ng/mL and 82.1 mg/L, respectively. The patient was diagnosed with necrotizing fasciitis which added to preexisting condition as a secondary pathology after extravasation injury get worse in time. So, an operation was scheduled. Aggressive surgical intervention, including debridement of all necrotic tissues and surrounding fibrotic, scarred skin and muscle in transition zone extending from the right neck to the right arm was carried out and tissue cultures were obtained (Figure 2). The wound was left open after irrigation and the patient was followed by dressing changes. A week later, the wound was covered with a split thickness skin graft. The deep tissue culture results obtained intraoperatively were negative.

## 3. Discussion

Necrotizing fasciitis is a serious infectious disease for which accurate diagnosis is often difficult at the initial presentation, which may result in a severe complications such as multiorgan failure and death. Predisposing factors and conditions include an older age, chronic illness (such as alcoholism and diabetes mellitus) and male gender, as well as possibly underlying immune deficiency. Some antineoplastic drugs such as Idarubicin may cause severe tissue damage and even necrosis upon leakage into subcutaneous tissue. These substances are known as vesicants [5].

In the presented case necrotizing fasciitis diagnosis was made by patient's clinical condition and the laboratory findings. Because necrotizing fasciitis needs early and aggressive debridement of all affected tissues, the results of tissue culture could not be waited for surgery decision and deep tissue cultures obtained in the operation were also negative. This does not mean that necrotizing fasciitis occurs without infection in the extravasation injuries. Negative culture results in this case were mostly related to that the patient was under intensive, strong antibiotic suppression during the presentation time to our clinic.

The treatment of necrotizing fasciitis resulting from extravasation injury is similar to the treatment of necrotizing fasciitis caused by other reasons. Early and wide debridement of all necrotic tissues, antibiotic therapy, and fluid replacement is carried out. However, decisionmaking may be challenging for surgeons when nonnecrotic, but intensely scarred tissues with decreased blood supply, is to be removed. Our approach to necrotizing fasciitis resulting from extravasation injury involved debridement of all indurated and fibrotic skin and muscles, in order to avoid further complications such as new infections or reoperations.

The purpose of this paper is to draw the attention of medical staff to the important effects of extravasation injury especially in immune-compromised patients. We suggest an early surgical debridement of all necrotic and fibrotic tissues that are affected by extravasation injury before necrotizing fasciitis develops, especially in immunocompromised individuals. If necrotizing fasciitis occurs in extravasation injury area, we advise a wide and aggressive surgical debridement of all necrotic, infected areas, including scar tissue at the periphery, in order to prevent morbidity and even mortality.
